# Splenectomy for solitary splenic metastasis of ovarian cancer

**DOI:** 10.1186/1471-2407-4-96

**Published:** 2004-12-22

**Authors:** Yang Seok Koh, Jung Chul Kim, Chol Kyoon Cho

**Affiliations:** 1 Department of Surgery, Chonnam University Medical School, Chonnam National University Hospital, 8 Hak-dong, Dong-gu, Gwangju 501-757, Korea

## Abstract

**Background:**

Splenic metastases occur in rare cases with a few case reports of patients in the literature. Generally, splenic metastases mean late dissemination of a disease. Solitary splenic metastases from solid tumors are extremely unusual.

**Case presentation:**

We report a case of a patient with ovarian mucinous cystadenocarcinoma who underwent splenectomy for isolated parenchymal metastasis.

**Conclusion:**

Ovarian epithelial tumors comprised most of isolated splenic metastases from gynecologic tumor. When isolated splenic recurrence is suspected on image studies and serum tumor markers, intraabdominal gross findings should be examined to exclude peritoneal carcinomatosis. If only spleen was under suspicion of recurrence of ovarian cancer, splenectomy may play a therapeutic role.

## Background

Splenic metastases from solid tumors occur in late stage of a disease, so those can hardly be an indication for a surgery. Cancers of the ovaries, lung, breast, stomach, skin and colon are known to metastasize to the spleen [1]. Even though recent reports suggest increasing incidence of splenic metastasis from gynecologic tumors [2-8], the number of the cases that isolated splenic metastasis is fewer than 25 in the literature worldwide. Among them, solitary parenchymal metastasis would comprise the small portion. Fewer than 15 cases of splenic metastasis occur from the ovaries as a primary site, pathology revealed cystadenocarcinoma.

We present a case of mucinous cystadenocarcinoma that recurred in the splenic parenchyma.

## Case presentation

A 29-year-old woman was admitted our hospital on August 1999, had been diagnosed as mucinous tumor of borderline malignancy a year ago. She was followed up at a local clinic. On the follow-up study, the patient's CEA level was raised to 43.32 U/L and CT scan showed splenomegaly with cystic lesion. Her past medical history was not significant. She already underwent two surgeries. The first surgery, right salpingo-oophorectomy, was performed at the age of 22 after being diagnosed as dermoid cyst. She was healthy thereafter. Seven years later, left ovarian mass was found on the routine check. At the second surgery, left ovarian mass excision, mucinous tumor of borderline malignancy was diagnosed.

**Figure 1:**
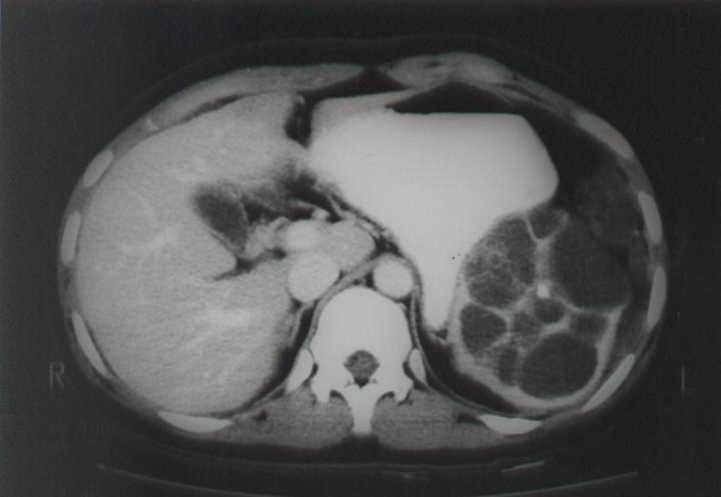
Abdomen CT shows 9 × 8 cm-sized multiseptated cystic lesion with inner calcification in the spleen.

During follow-up after the second surgery, she was referred to our hospital on the suspicion of carcinomatosis peritonei. On the preoperative evaluation (Figure 1), splenic lesion, which had been existed for 2 years, was merely noticed as simple cystic lesion unrelated to the ovarian mass.

To exclude peritoneal carcinomatosis, open laparotomy was perfomed. On opening the abdomen, no abnormal gross findings were found except the splenic lesion, which reported as probable metastatic adenocarcinoma on frozen sections. After splenectomy was carried out, peritoneal washings and multiple biopsies on the omentum, peritoneum, mesentery, and left ovary were performed to rule out possible microscopic peritoneal dissemination. Suspecting the transabdominal metastasis, 100 mg of cisplatin was infused into the peritoneal cavity at the end of the operation; however no intraperitoneal recurrence was confirmed after tissue diagnosis.

**Figure 2:**
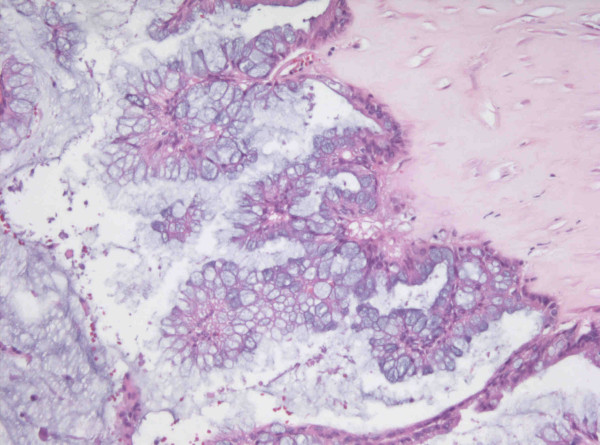
The section reveals papillary hyperplasia of mucinous lining epithelium with stratification and atypism (H&E stain, ×250).

Final pathologic examination (Figure 2) showed metastatic mucinous cystadenocarcinoma. Peritoneal washings and multiple biopsies were all negative. The patient was recovered from the surgery without the evidence of sepsis of severe thrombocytosis. The patient received 5 courses of Taxol and Carboplatin as postoperative chemotherapy.

Two years after the surgery, 3 × 3-sized mass on the left ovary, which assumed to be recurrence, was detected. She has been followed up at outpatient department receiving symptomatic treatment and chemotherapy.

## Discussion

The frequency of splenic metastasis has been reported 2.3 to 7.1 per cent from autopsy series of cancer patients [9-11]. Splenic metastases from the ovaries, uterus, uterine cervix, lung, breast, stomach, skin and colon have been reported, and ovarian cancer comprises the three fourth.

Until now, in the literature, splenic capsular metastasis has been reported to occur in the case of far advanced stage of a disease or in the case of more than one organ was already involved in terminal stage and especially in the case of peritoneal metastasis. When one or more organ in the thoracic cavity and abdominal cavity was involved, the rate of splenic metastasis was 43% [12]. But isolated splenic metastasis is rare; only fewer than 25 cases were reported. Some reported hematogenous metastasis to the parenchyma of the spleen [13]. This mean the spleen is the organ of the privilege [11,14,15]. It could be explained by hypothesis of the role of the splenic capsule as physical barrier; the lack of afferent lymphatics in the splenic parenchyma, the acute angle of the origin and the tortuosity of the splenic artery, the rhythmic contractile properties of the spleen, and the immune competence and possible antineoplastic nature of the splenic tissue itself [13]. In this case, solitary splenic parenchymal metastasis from ovarian epithelial tumor was made hematogenously without intraabdominal dissemination.

Among cases of the isolated splenic metastasis, the ovarian cancers make up the most. After Minazawa et al [16] firstly performed splenectomy in the splenic metastasis of ovarian cancer patient, splenectomy, as a therapeutic modality of splenic metastasis, was supported by similar articles and studies [17,18]. Recently, splenectomy was often included in the cytoreductive surgery of the ovarian cancer [2,3,19].

CT scanning and measurement of serum tumor markers, especially CA 125, are helpful for detecting the recurrence and the infrequent splenic metastasis.

We proposed that splenectomy be a proper therapeutic modality for an isolated splenic metastasis, especially parenchymal metastasis, from an ovarian cancer. When isolated splenic recurrence is suspected on the CT scanning and serum tumor markers, intraabdominal gross findings should be examined meticulously. If only spleen was under suspicion of recurrence, splenectomy would be a proper therapeutic procedure.

## Competing interests

The author(s) declare that they have no competing interests.

## Authors' contributions

YS carried out the literature search and prepared the manuscript. JC Kim, the corresponding author, was the main operator in charge of the case. CK was involved in the operation and the patient active management. Three authors read and approved the final manuscript.

## Pre-publication history

The pre-publication history for this paper can be accessed here:
